# Contribution of direct InhA inhibitors to novel drug regimens in a mouse model of tuberculosis

**DOI:** 10.1128/aac.00357-24

**Published:** 2024-09-30

**Authors:** Lourdes Encinas, Si-Yang Li, Joaquin Rullas-Trincado, Rokeya Tasneen, Sandeep Tyagi, Heena Soni, Adolfo Garcia-Perez, Jin Lee, Rubén González del Río, Jaime De Mercado, Verónica Sousa, Izidor Sosič, Stanislav Gobec, Alfonso Mendoza-Losana, Paul J. Converse, Khisi Mdluli, Nader Fotouhi, David Barros-Aguirre, Eric L. Nuermberger

**Affiliations:** 1 Global Health Medicines R&D, GSK, Tres Cantos, Madrid, Spain; 2 Center for Tuberculosis Research, Division of Infectious Diseases, Johns Hopkins University, Baltimore, Maryland, USA; 3 Discovery DMPK, GSK, Tres Cantos, Madrid, Spain; 4 Faculty of Pharmacy, University of Ljubljana, Ljubljana, Slovenia; 5 TB Alliance: Global Alliance for Tuberculosis Drug Development, New York, New York, USA; St. George's, University of London, London, United Kingdom

**Keywords:** InhA inhibitor, tuberculosis, mouse, GSK138, GSK3081138A, GSK693, GSK2505693A, tuberculosis drugs

## Abstract

Isoniazid is an important first-line medicine to treat tuberculosis (TB). Isoniazid resistance increases the risk of poor treatment outcomes and development of multidrug resistance, and is driven primarily by mutations involving *katG*, encoding the prodrug-activating enzyme, rather than its validated target, InhA. The chemical tractability of InhA has fostered efforts to discover direct inhibitors of InhA (DIIs). In this study, we bridge the gap in understanding the potential contribution of DIIs to novel combination regimens and demonstrate a clear distinction of DIIs, like GSK693 and the newly described GSK138, from isoniazid, based on activity against clinical isolates and contribution to novel drug regimens. The results suggest that DIIs, specifically GSK138 and GSK693, could be promising partners in novel drug regimens, including those used against isoniazid-resistant TB, potentially enhancing their efficacy and/or preventing the selection of resistant mutants and supporting the continued exploration of InhA as a promising target for TB drug development.

## INTRODUCTION

Tuberculosis (TB) is a communicable disease caused by *Mycobacterium tuberculosis* (*M.tb*). Globally, an estimated 10.6 million people developed TB in 2022, up from best estimates of 10.3 million in 2021 and 10.0 million in 2020 (1). With timely diagnosis and treatment with first-line drugs, most people who develop TB are cured, and onward transmission of infection is curtailed.

Isoniazid (H), a prodrug activated by the mycobacterial catalase-peroxidase enzyme KatG (2), is an important first-line TB drug. Baseline isoniazid resistance increases the risk of suboptimal treatment outcomes (e.g., treatment failure or relapse) and acquisition of multidrug-resistant (MDR) TB. Based on evidence reviews indicating reduced efficacy of the standard first-line drugs for the treatment of isoniazid-resistant tuberculosis (Hr-TB) (3–8), the World Health Organization issued a supplement to its guidelines for the treatment of drug-resistant TB in 2018, providing new recommendations for the management of Hr-TB (9). Resistance to isoniazid is primarily caused by mutations in the activating enzyme KatG or in the upstream promoter region of the gene encoding the target of isoniazid, InhA. Only rarely do resistance mutations occur in the InhA enzyme itself. Combinations of these mutations may also occur. By and large, the most common mutations in Hr-TB strains are found in *katG* and confer “high-level” resistance, even in the absence of an *inhA* mutation. In this situation, the inclusion of isoniazid in the regimen, even at high doses, is unlikely to increase its effectiveness (10). On the other hand, mutations in the *inhA* promoter or in the *inhA* gene are generally associated with lower-level resistance than *katG* mutations, and higher doses of isoniazid (10–15 mg/kg/day) may result in bactericidal activity against such *inhA* mutants similar to that observed with standard isoniazid doses (4–6 mg/kg/day) against fully susceptible strains, especially in individuals with slow or intermediate isoniazid acetylation phenotypes (2, 11–13).

The opportunity to overcome the high rate of clinical resistance to isoniazid due to *katG* mutations, together with the biological relevance of InhA (target validated clinically by isoniazid and ethionamide) and its chemical tractability (14), has fostered efforts to discover direct inhibitors of InhA (DIIs). In recent years, three structurally different DIIs such as NITD-916 (15), GSK2505693A (GSK693) (16), and AN12855 (17) have demonstrated *in vivo* efficacy in murine TB models upon oral administration, offering a potential strategy to circumvent isoniazid resistance mediated through *katG* mutations.

To our knowledge, GSK693 was the first DII to demonstrate *in vivo* efficacy comparable to that of isoniazid (16). More recently, Xia and co-authors reported the discovery of a direct, co-factor-independent inhibitor of InhA, AN12855, which showed good efficacy in acute and chronic murine TB models that was also comparable to isoniazid (17, 18). However, the efficacy of DIIs in combination with other TB drugs has not been reported. Moreover, the high preliminary human dose prediction of GSK693 hampers its further development as a lead compound (data not published). GSK3081138A (GSK138), another compound within the same thiadiazole-based series as GSK693, was identified as a promising back-up compound for further study.

The objectives of the current study were to explore the therapeutic potential of DIIs such as GSK693 and GSK138 by assessing their *in vitro* activity against clinical isolates and evaluating their *in vivo* efficacy in combination with new and existing TB drugs, particularly in the context of regimens used to treat isoniazid-resistant TB.

## RESULTS

GSK138 is similar in structure to GSK693. It is a slightly more lipophilic compound with a well-balanced profile of physicochemical properties, *in vitro* potency, *in vivo* pharmacokinetics (PK), and safety (Table 1), which support its progression for further investigation.

**TABLE 1:** Structure and properties of the optimized lead **GSK138**^*b*^

		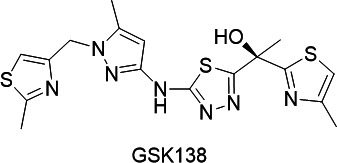
Physicochemical properties	MW	433
	clogP/Chrom logD	1.2/3.39
	Permeability Papp (Madin-Darby canine kidney-MDR1)	374 nm/s
	Solubility fasted state simulated intestinal fluid (pH 6.5)	140–320 μM
Activity profile	InhA IC_50_^*a*^	0.04 µM ± 0.01
	*Mtb* MIC	1 µM
	*Mtb* intracell MIC^*a*^	0.9 µM ± 0.1
Cytotoxicity profile	HepG2 cytotoxicity Tox_50_	>100 µM
	Cell health IC_50_ (nuclear size, mitochondrial membrane potential, and plasma membrane permeability)	>199.5 µM
Genetic toxicity assessment	Ames test	Negative
Cardiovascular profile	hERG Qpatch IC_50_	>30 µM
Microsomal stability assessment	*In vitro* Clint mouse/rat/dog/human	4.4-/3.5-/1.7-/0.3-mL/min/g tissue
*In vivo* pharmacokinetic profile	*In vivo* Cl mouse (1 mg/kg i.v.)^*a*^	77.6 ± 16.8 mL/min/kg
	*In vivo* Vss mouse (1 mg/kg i.v.)^*a*^	2.6 ± 0.3 L/kg

^*a*^ Mean ± standard deviation.

^*b*^ The lead was assessed for activity against *M. tuberculosis* H37Rv both intracellularly and extracellularly. The physicochemical and absorption, distribution, metabolism, elimination, and toxicity (ADMET) properties were determined as well. i.v., intravenous; MIC, minimum inhibitory concentration.

GSK138 is a medium molecular weight compound with a chrom logD at pH 7.4 of 3.38. The measured solubility in fasted state simulated intestinal fluid (FaSSIF) was high (Table 1). The permeability of GSK138 predicted from Madin-Darby canine kidney (MDCK) cells was also high (Table 1). The efflux ratio determined by assays with and without incubation with a potent P-glycoprotein (P-gp) inhibitor indicated that it is a P-gp substrate.

GSK138 inhibited recombinant InhA with an IC_50_ of 0.04 µM. The minimum inhibitory concentration (MIC) was 1 µM against *M. tuberculosis* H37Rv, and GSK138 retained its activity against intracellular bacteria growing inside THP-1-derived macrophages *in vitro* (MIC 0.9 µM). Additionally, it showed no effect up to the highest concentration tested (200 µM) in the cell health assay (measuring membrane, nuclear, and mitochondrial damages). The preliminary toxicological profile showed an overall clean *in vitro* safety profile.

To assess the susceptibility of GSK138 to P450-mediated phase I metabolism, metabolic stability was determined during incubation in CD1 mouse, Sprague Dawley rat, beagle dog, and human liver microsomes. GSK138 exhibited moderate *in vitro* clearance in liver microsomes from the pre-clinical species, and low *in vitro* clearance in humans.

To determine the pharmacokinetic parameters, GSK138 was administered intravenously [formulation: 5% dimethyl sulfoxide (DMSO)/20% Encapsin in saline solution] as a single bolus dose in C57BL/6 mice at a target dose of 1 mg/kg. All pharmacokinetic parameters were determined in whole blood (Table 1). A moderate clearance and a moderate volume of distribution were observed.

The minimum concentrations of GSK693 and GSK138 that inhibit 90% of isolates tested (MIC_90_) were determined against a set of drug-susceptible, MDR, and extensively drug-resistant (XDR) *M.tb* clinical isolates. Both GSK693 and GSK138 retained activity against these clinical isolates (GSK693 MIC_90_ = 1.87 µM, GSK138 MIC_90_ = 3.75 µM), similar to the MICs against strain H37Rv in the same assay (Table S1). As expected for DIIs, the thiadiazole compounds have KatG-independent activity. No change in MIC was observed against isonazid-resistant clinical isolates carrying mutations in *katG* S315T. Clinical isolates carrying an *inhA* C-15T mutation have increased InhA production which confers low-level resistance to isoniazid. Among eight clinical isolates with the *inhA* C-15T mutation, two showed low-level resistance to the thiadiazoles (i.e., MICs for both DIIs between 4 and 16 times the MIC of the susceptible reference strain), and one showed moderate-level resistance (>16 times to 80 times the MIC of the susceptible reference strain). Thiadiazoles remained equally effective among the rest of the sensitive, MDR, and XDR *M.tb* clinical isolates tested.

**Fig 1:**
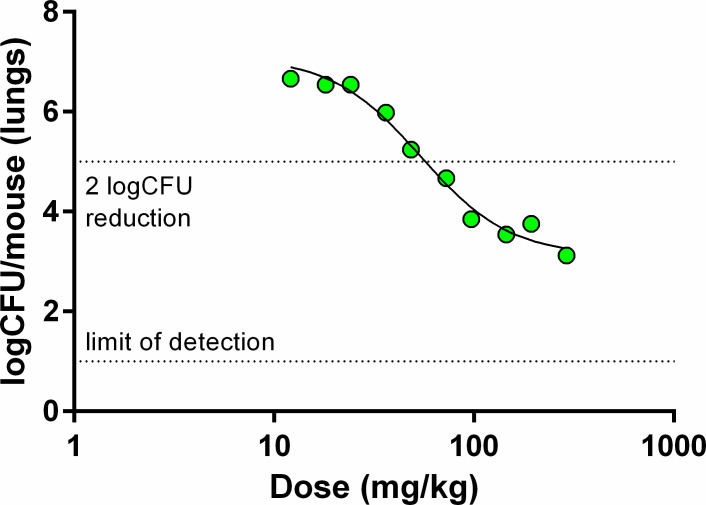
Dose-response relationship for GSK138 in an acute mouse infection model of TB. Each point represents data from an individual mouse that received GSK138 administered orally once daily for 8 days.

Based on GSK138’s overall profile, the therapeutic efficacy of GSK138 against *M.tb* in an acute murine model of intratracheal infection was determined (see Fig. 1).

This acute infection model measures antitubercular activity on fast-growing bacteria (19). Treatment is administered for 8 consecutive days, starting 1 day after intratracheal infection with approximately 10^5^ colony-forming units (CFUs). Because the bacterial load reduction previously observed with GSK693 was similar in both acute and chronic murine TB models (16), we performed a full dose-response study only in the acute model to characterize the compound and estimate the optimal dose for future combination studies. GSK138 induced a net killing of the bacteria at the highest doses. The ED_99_ (the dose producing a 2-log_10_ reduction in CFUs compared to untreated control mice) for GSK138 was 57 mg/kg [95% confidence interval (CI): 50–67 mg/kg] and the dose of GSK138 at which 90% of the maximum bactericidal effect was achieved (ED_max_) was 167 mg/kg (95% CI: 125 to >290 mg/kg). The whole blood area under the concentration-time curve over 24 h post-dose (AUC_0–24 h_) at steady-state associated with this ED_max_ (AUC_EDmax_) was 68,544 ng·h/mL. Comparison with previous data suggests that GSK138 is as efficacious as GSK693 at a lower exposure, and therefore, GSK138 has the potential for a lower-dose prediction in humans.

**Fig 2:**
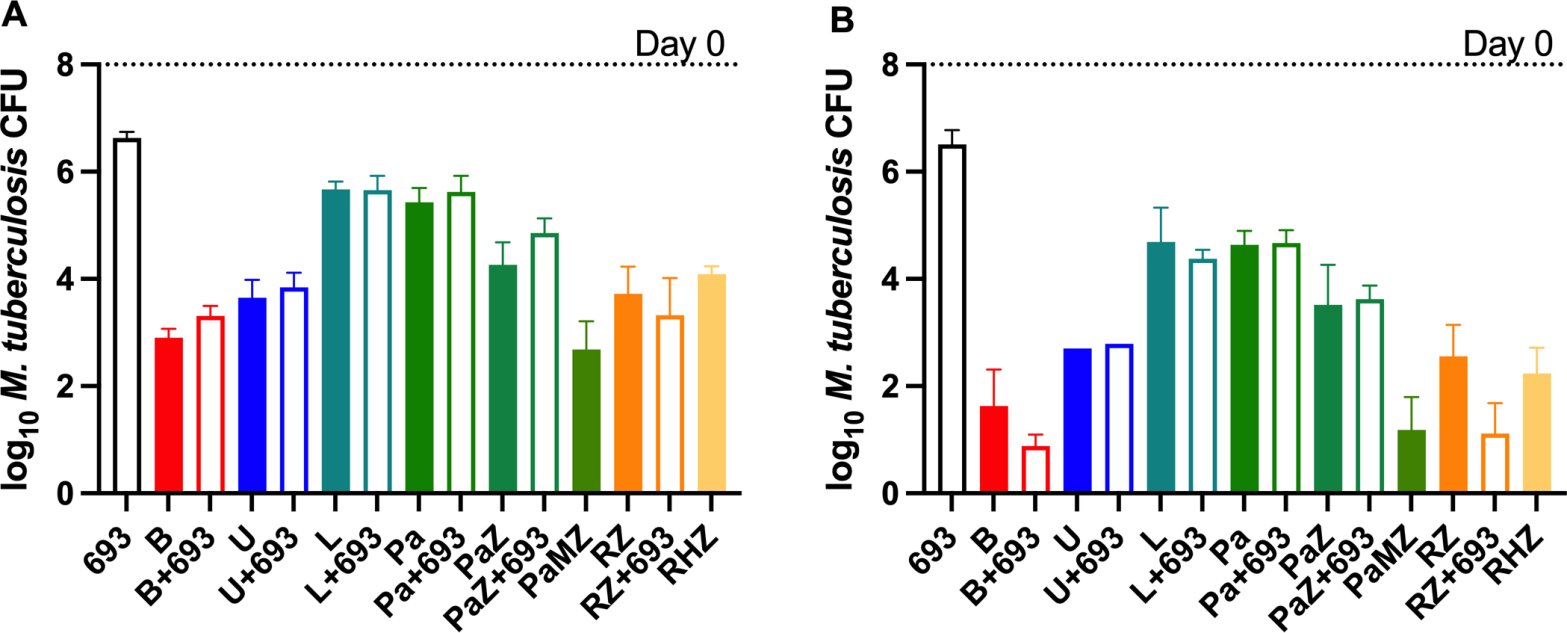
Mean (±SD) lung CFU counts at D0 and after 4 (**A**) or 8 (**B**) weeks of treatment in experiment 1. In combination with RZ, but not with other drugs, GSK693 showed significantly enhanced antibacterial activity at week 8 (**B**) but not at week 4 (**A**). Open bars show lung CFU counts with the addition of GSK693 to drugs, shown in solid bars. Drug doses: R, 10 mg/kg; Z, 150 mg/kg; H, 10 mg/kg; 693, 300 mg/kg; Pa, 50 mg/kg; M, 100 mg/kg; B, 25 mg/kg; L, 100 mg/kg; and U, 50 mg/kg. 693, GSK693; B, bedaquiline; H; isoniazid; L, linezolid; M, moxifloxacin; Pa, pretomanid; R, rifampicin; RZ, rifampicin and pyrazinamide; U, sutezolid; Z, pyrazinamide

Ultimately, any antitubercular drug must be used in combination with other antitubercular drugs to treat active TB. The success of any new regimen will depend on the properties of these drugs and how they work in combination. GSK693 and GSK138 showed suitable profiles to justify investigation of the efficacy of these DIIs in combination with other drugs in animal models. Firstly, GSK693 was selected as a tool compound to learn about the chemical series and its interactions with potential companion drugs. Experiment 1 was performed in a well-established high-dose aerosol infection model (20) with the following objectives: (i) to evaluate its ability to replace H in combination with rifampicin (R) and pyrazinamide in the core first-line regimen; (ii) to evaluate its ability to replace moxifloxacin (M) in combination with pretomanid and pyrazinamide in the novel PaMZ regimen; and (iii) to evaluate its contribution to the bactericidal activity of two-drug combinations including bedaquiline, sutezolid, linezolid, and pretomanid (Fig. 2).

In this model, in which untreated mice routinely succumb to infection with lung CFU counts above 8 log_10_ within the first 3–4 weeks after infection, GSK693 (300 mg/kg) reduced the lung CFU counts by 1.34 and 2.33 log_10_ after 4 and 8 weeks of treatment, respectively. No additive effect was observed when GSK693 was combined with sutezolid, linezolid, or pretomanid, nor was it as effective as moxifloxacin in combination with pretomanid and pyrazinamide. However, the combination of GSK693 with rifampicin and pyrazinamide (RZ) was significantly more active than RZ alone or in combination with isoniazid after 8 weeks of treatment (*P* < 0.05). Notably, compared to bedaquiline monotherapy, the combination of GSK693 with bedaquiline resulted in reduced bactericidal activity after 4 weeks of treatment (*P* = 0.02) but greater activity after 8 weeks of treatment (*P* = 0.08). The additive effect of GSK693 at week 8 was attributable to its prevention of selection of bedaquiline-resistant mutants, as emergence of bedaquiline resistance was observed in two of the four mice treated with bedaquiline alone for 8 weeks, consistent with previous results (21). Excluding these two mice from the analysis revealed no difference between treatment with bedaquiline alone and bedaquiline plus GSK693**.**

The promising result observed with RZ + GSK693 (in experiment 1) prompted a follow-up experiment to confirm the additive effect of GSK693, evaluate the dose-response relationship for GSK693, and explore potential drug-drug interactions in the RZ + GSK693 combination.

**Fig 3:**
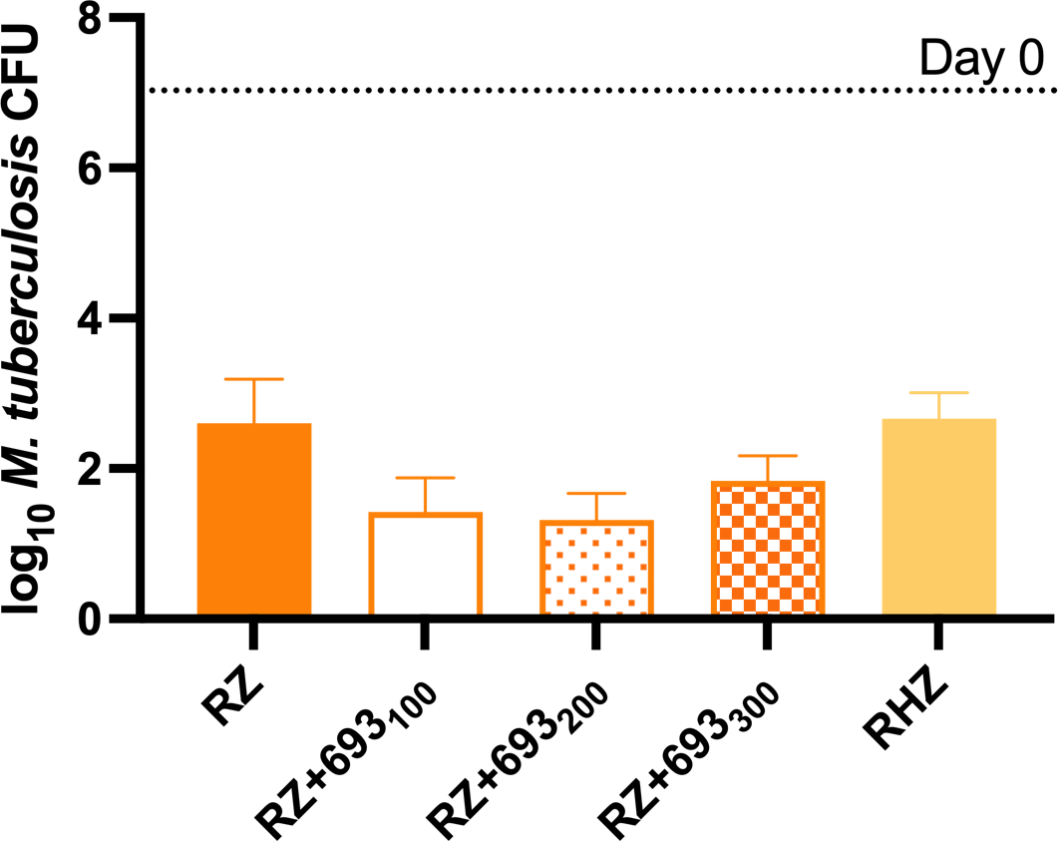
Mean (±SD) lung CFU counts at D0 and after 8 weeks of treatment in experiment 2. GSK693 significantly enhanced, in a non-dose-dependent manner, the activity of the RZ (rifampicin, 10 mg/kg, plus pyrazinamide 150 mg/kg) combination. Isoniazid (10 mg/kg) did not enhance the activity of the combination. GSK693 dose (in mg/kg) is indicated in subscripts. 693, GSK693.

As observed in experiment 1, the addition of GSK693, but not isoniazid, significantly increased the activity of the RZ combination in experiment 2 (Fig. 3).

The magnitude of the additive effect was also similar between experiments. The addition of GSK693 at 300 mg/kg to RZ reduced the lung CFU counts by an additional 1.44 log in experiment 1, as compared to a reduction of 0.76 log (*P* < 0.05 vs RZ) in experiment 2. Remarkably, however, greater reductions of 1.17 and 1.28 log (*P* < 0.01 and 0.001 vs RZ, respectively) were observed when GSK693 was used at 100 and 200 mg/kg, respectively, in experiment 2. The mean lung CFU count in the RZ + GSK693 arm at week 8 was 1.12 log_10_ lower than that in the RHZ arm (*P* = 0.02) in experiment 1 and ranged from 0.83 to 1.35 log_10_ lower than RHZ (*P* > 0.03), depending on the GSK693 dose, in experiment 2.

Although the sparse sampling prevented a formal assessment of the PK profile, the apparent lack of GSK693 dose-response was not explained by the GSK693 exposures at the 100-, 200-, and 300-mg/kg doses (Table 2).

**TABLE 2:** Data obtained from monocompartmental model of sparse plasma sampling concentrations in the combination study^*a*^

GSK693 dose (mg/kg)	Plasma AUC_0–24 h_ (ng·h/mL)	Blood AUC_0–24 h_ (ng·h/mL)
100	12,867	23,032
200	27,337	48,933
300	64,866	116,110

^*a*^ A blood/plasma ratio of 1.79 was used to transform the plasma parameters to blood values.

Interestingly, the observed effect of GSK693 in combination was achieved at a lower exposure than that needed to achieve the maximum effect in monotherapy in the acute infection model (110,200 ng·h/mL). Based upon the potential for drug-drug interactions, rifampicin was administered 1 h prior to other drugs (22). Plasma exposure of rifampicin (AUC_0–24 h_ = 68,544 ng·h/mL) when co-administered with pyrazinamide showed no evidence of a higher exposure that could explain the increase in the efficacy of the combination when compared to prior data for rifampicin when co-administered with pyrazinamide (AUC_0–24 h_ = 160,600 ng·h/mL) (22) or as monotherapy at 10 mg/kg (AUC_0–24 h_ = 87,200–142,100 ng·h/mL) (23).

The result from the combination of GSK693 with RZ proved to be superior to the first-line treatment (RHZ). This result encouraged further combination experiments, now with GSK138.

**Fig 4:**
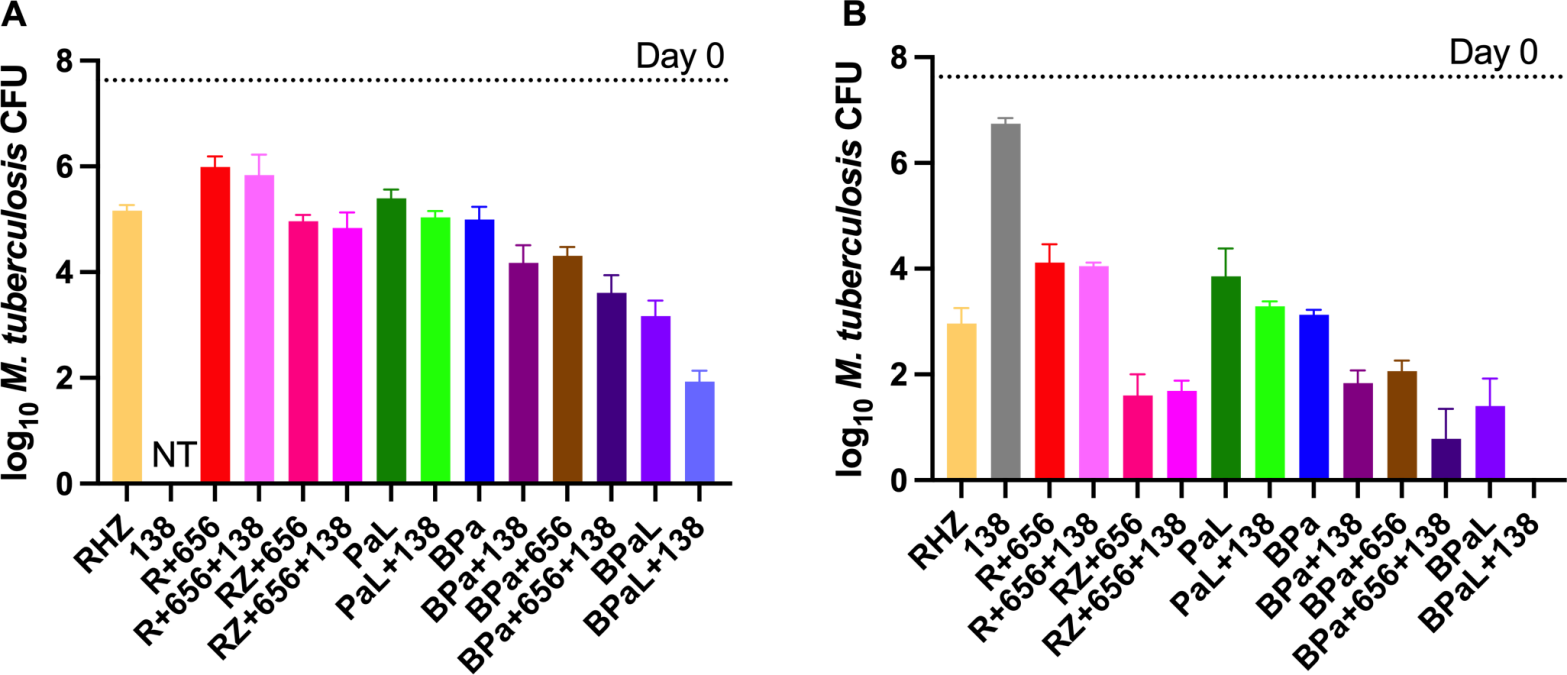
The direct InhA inhibitor GSK138 enhanced the activity of the BPa, BPaL, and BPa + GSK656 combinations after 4 weeks (**A**) or 8 weeks (**B**) of treatment in experiment 3. After 8 weeks of treatment, the BPaL + GSK138 regimen rendered mouse lung cultures negative. Data are presented as mean (±SD) lung CFU counts. R, 10 mg/kg; Z, 150 mg/kg, H, 10 mg/kg; 138, 200 mg/kg; 656 (sulfate salt), 10 mg/kg; Pa, 50 mg/kg; B, 25 mg/kg; L, 100 mg/kg. 138 = GSK138; 656, GSK656; B, bedaquiline; H, isoniazid; L, linezolid; NT, not tested; Pa, pretomanid; R, rifampicin; Z, pyrazinamide.

A major objective of experiment 3 was to determine the effect of adding GSK138 to the novel regimen of bedaquiline, pretomanid, and linezolid (BPaL) recently approved for the treatment of XDR and treatment-intolerant or non-responsive MDR TB and the effect of substituting GSK138 for either bedaquiline or linezolid. The experiment also included the novel LeuRS inhibitor GSK3036656 (GSK656) (24, 25) that is now in phase 2 clinical trials. The objectives of this experiment were the following: (i) to evaluate the contribution of GSK138 to the efficacy of three- and four-drug combinations based on the BPa backbone, and (ii) to evaluate the effect of adding GSK138 to the combination of rifampicin plus GSK656, with or without pyrazinamide (Fig. 4).

The addition of GSK138 to BPaL, its BPa backbone, or the novel BPa +GSK656 regimen significantly increased the activity of each combination after 4 weeks (*P* < 0.01) and after 8 weeks (*P* < 0.0001) of treatment. Indeed, the four-drug combination of BPaL plus GSK138 was the only regimen tested to render all mice culture-negative after 8 weeks of treatment. After 8 weeks of treatment, the activity of the three- and four-drug regimens containing BPa plus GSK138, with or without GSK656, was statistically indistinguishable from that of BPaL and significantly superior to the first-line RHZ regimen (*P* < 0.0001). The addition of GSK138 did not significantly increase the activity of PaL, R + GSK656, or RZ + GSK656.

**Fig 5:**
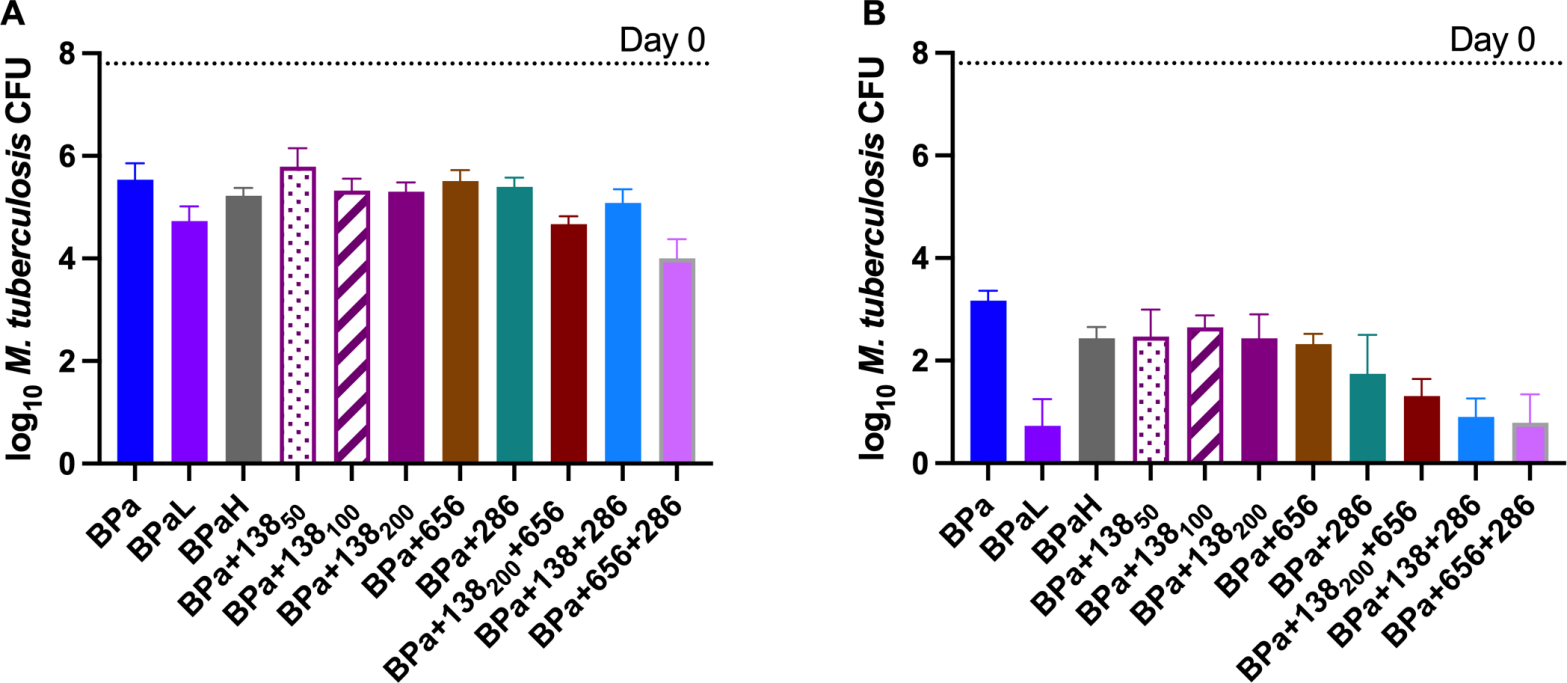
GSK138 significantly enhanced the activity of BPa and BPa-based regimens at 4 weeks (**A**) or 8 weeks (**B**), particularly in combination with GSK286. In combination with GSK656 or GSK286, the 200-mg/kg dose of GSK138 was used. Data are presented as mean (±SD) lung CFU counts. B, 25 mg/kg; Pa, 100 mg/kg; L, 100 mg/kg; H, 10 mg/kg; 286, 50 mg/kg; 656 (hydrochloride salt), 10 mg/kg. GSK138 dose (in mg/kg) is indicated in subscripts. 286, GSK286; 656, GSK656; B, bedaquiline; H, isoniazid; L, linezolid; Pa, pretomanid.

Experiment 4 (Fig. 5) was performed to confirm the contribution of GSK138 to the BPa backbone, this time including a range of GSK138 doses. The experiment also included isoniazid as a comparator and combinations with GSK656 and the novel cholesterol-dependent inhibitor GSK2556286 (GSK286) (26), which is currently being investigated in a first time-in-human study to evaluate its safety, tolerability, and pharmacokinetics (NCT04472897).

The results reaffirmed the additive effects of GSK138 when added to BPa for 8 weeks of treatment (*P* < 0.05 for BPa + GSK138 at 50 or 200 mg/kg vs BPa at week 8). Similar results were observed with the addition of isoniazid or GSK656 to BPa (*P* = 0.0002 and *P* < 0.0001, respectively). No dose-response of GSK138 was evident after 8 weeks, and unlike in experiment 3, BPa plus GSK138 was less effective than BPaL (*P* < 0.01 after 4 and 8 weeks). However, as observed in experiment 3, the additive four-drug combination of BPa + GSK656 with GSK138 was statistically indistinguishable from BPaL, as was the combination of BPa + GSK286 with GSK138. These four-drug combinations of BPa + GSK138 plus either GSK656 or GSK286 also had bactericidal activity similar to BPa + GSK656 + GSK286 after 8 weeks of treatment.

**Fig 6:**
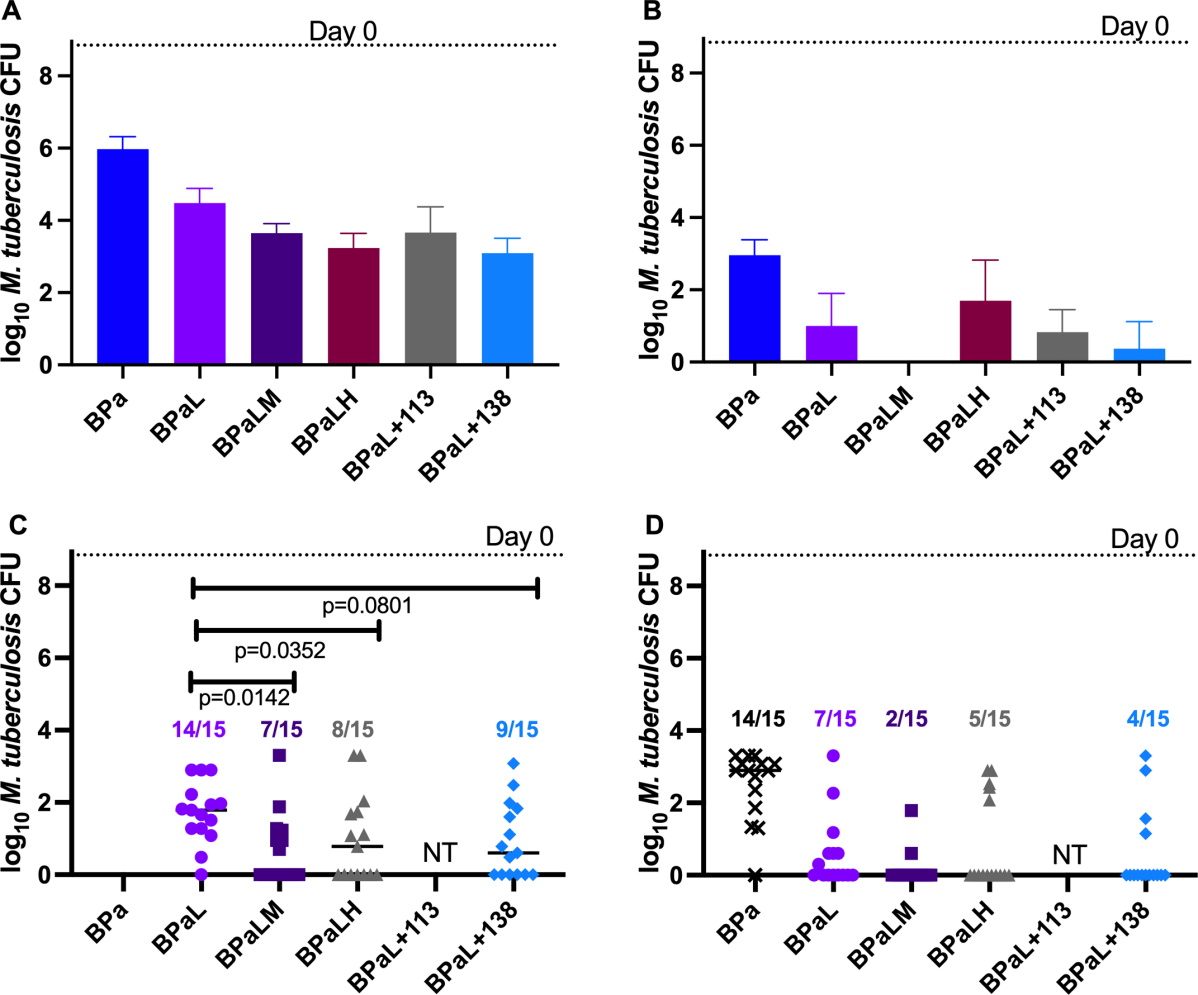
The addition of an InhA inhibitor or moxifloxacin enhanced the bactericidal and sterilizing activity of the BPaL regimen. After both 4 weeks (**A**) and 8 weeks (**B**), the addition of moxifloxacin, isoniazid, NITD-113, or GSK138 to BPaL enhanced the bactericidal activity compared to BPaL alone (with the exception of isoniazid at week 8). Data are presented as mean (±SD) lung CFU counts. The proportion of mice relapsing after 8 weeks of treatment, followed by 12 weeks of no treatment (**C**), was statistically significantly lower in the presence of either moxifloxacin or isoniazid and approached statistical significance with GSK138. There were fewer relapses after 12 weeks of treatment and 12 weeks of follow-up (**D**) with the addition of a fourth drug, although these differences were not statistically significant. The proportions of mice relapsing are indicated above the symbols for lung CFU counts. B, 25 mg/kg; Pa, 100 mg/kg; L, 100 mg/kg; M, 100 mg/kg; H, 10 mg/kg; NITD-113 (see Introduction), 150 mg/kg; 138, 200 mg/kg. 138, GSK138; B, bedaquiline; H, isoniazid; L, linezolid; M, moxifloxacin; NITD-113, prodrug for NITD-916; NT, not tested; Pa, pretomanid.

Given the superior bactericidal activity of the BPaL plus GSK138 regimen compared to BPaL alone in experiment 3, experiment 5 was performed to determine whether addition of GSK138 to BPaL could shorten the duration of treatment needed to prevent relapse (Fig. 6). Comparator regimens included BPaL plus one of the following: isoniazid, NITD-113 (prodrug for NITD-916, a previously reported DII based on a different scaffold than GSK138) (15) and M. As observed in experiment 3, the addition of GSK138 at a dose of 200 mg/kg significantly increased the bactericidal activity of BPaL after 4 weeks of treatment (*P* < 0.01), as did isoniazid (*P* < 0.01), while there was a trend toward enhanced activity with NITD-113 (*P* = 0.11) and moxifloxacin (*P* = 0.10). BPaL + GSK138 resulted in fewer culture-positive mice and a lower mean CFU count after 8 weeks compared to BPaL and BPaL plus other InhA inhibitors, although these differences were not statistically significant (*P* = 0.09 for BPaL + GSK138 vs BPaL + isoniazid). Only the BPaLM regimen rendered all mice culture negative at this time point. Similarly, the addition of GSK138, isoniazid, or moxifloxacin to BPaL each reduced the proportion of mice relapsing after 8 and 12 weeks of treatment compared to BPaL alone, although the differences were statistically significant only after 8 weeks of treatment with moxifloxacin or isoniazid, as shown in Fig. 6C.

## DISCUSSION

The thiadiazole-based DIIs (namely, GSK693) proved capable of replacing isoniazid in the first-line regimen. In fact, the bactericidal activity of the regimen increased with this substitution, and addition of GSK693 increased the activity of the rifampicin-pyrazinamide combination. The mechanism responsible for the superior activity of the DII in this regimen requires further study, but superior killing of phenotypically isoniazid-tolerant persisters that have relatively lower *katG* expression, whether stochastically or in response to stress (27), is one possible explanation. Additional studies in a relapsing mouse model are an important next step to determine whether further development of thiadiazole DIIs could yield superior first-line regimens containing rifamycins and pyrazinamide.

The thiadiazole-based DIIs (namely, GSK138) also proved capable of increasing the bactericidal activity of BPa-based regimens and may increase the sterilizing activity as well, as the difference in proportions of mice relapsing after 8 weeks of treatment with BPaL + GSK138 vs BPaL missed achieving statistical significance by a single relapse event. BPaL is an effective 6-month all-oral regimen for XDR-TB and difficult-to-treat MDR-TB cases (28, 29). Use of BPaL + moxifloxacin is recommended when fluoroquinolone susceptibility is suspected, based on results of the TB-PRACTECAL trial (30). The improved efficacy observed with the addition of GSK138 to BPaL, which was qualitatively similar to that observed with the addition of moxifloxacin in experiment 5, suggests that this or another DII could provide an alternative to BPaL + moxifloxacin in case of fluoroquinolone resistance and improve the BPaL regimen by hastening the rate of sputum culture conversion, increasing the overall cure rate, shortening treatment duration, and/or reducing the emergence of drug resistance. Considering the strong overall activity of BPa + GSK138 and GSK693’s ability to prevent selection of bedaquiline-resistant mutants, the DIIs of this class could also reduce the need for linezolid, the most toxic component of the BPaL regimen allowing lower doses and/or shorter durations of linezolid.

The addition of GSK138 at 200 mg/kg consistently increased the bactericidal activity of BPa-based combinations in experiments 3–5. Lower doses of 50–100 mg/kg were less consistent in achieving significant reductions in CFU when added to BPa in experiment 4, although the addition of GSK138 at 50 mg did reduce the mean CFU after 8 weeks of treatment in a statistically significant manner. Further study of the exposure-response relationships for thiadiazole DIIs in combination with BPa is warranted.

The use of thiadiazole DIIs alone or in combination with GSK656 to replace linezolid entirely, if proven safe, could enable the use of BPa-based regimens as alternative, more universally active, first-line regimens that would be less affected by isoniazid monoresistance or MDR.

Although not the focus of this report, we observed that the addition of moxifloxacin to BPaL improved the bactericidal and sterilizing activity of the regimen. BPaL and BPaLM were studied in the TB-PRACTECAL trial (ClinicalTrials.gov Identifier: NCT02589782). Our results, which were obtained before the start of the trial, predicted superior efficacy of the four-drug combination. Indeed, a higher rate of sputum culture conversion at 8 weeks was observed in TB-PRACTECAL participants undergoing treatment with BPaLM vs BPaL (77% vs 46%) (30).

This research adds to the limited knowledge of the activity of direct InhA inhibitors in combination with new and existing TB drugs. The results suggest that a direct InhA inhibitor (e.g., GSK138 and GSK693) could be a promising partner in novel drug regimens, enhancing their efficacy and/or preventing the selection of bedaquiline-resistant mutants. These findings increase our understanding of the mechanism of action of direct inhibitors of InhA and provide further impetus to continue exploiting InhA as a promising target for TB drug development.

## MATERIALS AND METHODS

### Chemistry

A micromilling method was applied to GSK138 for particle size reduction in order to obtain a micronized GSK138 that was used in *in vivo* experiments. The Mixer Mill MM 301 (Retsch) was used at a frequency of 20 Hz for four cycles of 5 min.

NMR spectra were recorded on an Agilent Inova 600-MHz spectrometer equipped with a 5-mm Triple Resonance Gradient Probe IDTG600-5 (experiments run under software version vnmr3.2 Revision A). Measurements were made at room temperature in DMSO-d6 solvent. The chemical shift (*d*) values are expressed in parts per million (ppm), and coupling constants are in hertz. The chemical shifts (*δ*) were given relative to the residual ^1^H and ^13^C signals of the solvent peak as an internal standard: in ^1^H NMR (600 MHz) δ 2.49 ppm (quin, C_2_D_5_HOS) for DMSO-*d*_6_; in ^13^C NMR (150 MHz) δ 40.07 ppm (sept) for DMSO-*d*_6_ (legend: s, singlet; d, doublet; sept, septet; br, broad signal). Liquid chromatography-mass spectrometry (LC-MS) purity data were collected using a Waters Acquity UPLC instrument coupled with Waters Acquity single quadrupole mass and photodiode array detectors. High-resolution MS (HRMS) was performed on a QSTAR Elite System mass spectrometer. ^1^H NMR (600 MHz, DMSO-*d*_6_): δ 10.67 (s, 1H, NH), 7.26 (s, 1H, OH), 7.18 (s, 1H), 7.09 (s, 1H), 5.79 (s, 1H), 5.16 (s, 2H), 2.59 (s, 3H), 2.29 (s, 3H), 2.26 (s, 3H), and 1.97 (s, 3H); ^13^C NMR (150 MHz, DMSO-*d*_6_): δ 175.96, 166.46, 166.29, 164.69, 152.55, 152.26, 147.61, 140.88, 116.47, 115.38, 94.56, 74.68, 48.82, 28.98, 19.29, 17.51, and 11.53; and HRMS (ESI) *m*/*z*: calcd for C_17_H_19_N_7_OS_3_ [M + H]^+^, 434.0891; found, 434.0889.

### Permeability studies

Studies were performed as described by Polli et al. (31), with minor modifications. GF120918 was used as the inhibitor of P-gp. Apical-to-basolateral and basolateral-to-apical transports were studied across MDR1-MDCKII cell monolayers in the absence and presence of the P-gp inhibitor GF120918, and the Papp (intrinsic apparent permeability) was estimated in both directions with or without inhibitor.

### Solubility studies

Solubility assays were performed using a miniaturized shake flask method. Stock solutions (10 mM) of each test compound were used to prepare calibration standards (10–220 μM) in DMSO and to spike (1:50) duplicate aqueous samples of FaSSIF (simulating fasting state biorelevant media, pH 6.5) with a final DMSO concentration of 2%. After shaking for 2 h at 25°C, the solutions were filtered and analyzed by means of high-performance liquid chromatography with diode-array detection (HPLC-DAD) (Agilent 1200 Rapid Resolution HPLC with a diode array detector). Best-fit calibration curves were constructed using the calibration standards, which were used to determine the aqueous sample solubility (32).

### Bacterial strains

Tuberculosis H37Rv was mouse-passaged, frozen in aliquots, and subcultured in Middlebrook 7H9 broth with 10% oleic acid-albumin-dextrose-catalase (OADC) (Fisher, Pittsburgh, PA, USA) and 0.05% Tween 80 prior to high-dose mouse aerosol infection. MDR and XDR *M. tuberculosis* clinical isolates representing different resistance phenotypes belong to the collection of strains of the Vall d’Hebron Hospital of Barcelona.

*M. tuberculosis* H37Rv and H37Rv-Luc were routinely propagated at 37°C in Middlebrook 7H9 broth (Difco) supplemented with 10% Middlebrook albumin-dextrose-catalase (ADC)(Difco), 0.2% glycerol, and 0.05% (vol/vol) tyloxapol or on Middlebrook 7H10 agar plates (Difco) supplemented with 10% (vol/vol) OADC (Difco). Hygromycin B was added to the medium (50 µg/mL) to ensure plasmid maintenance when propagating the H37Rv-Luc strain. This strain constitutively expresses the luciferase *luc* gene from *Photinus pyralis* (GenBank accession number M15077) cloned in a mycobacterial shuttle plasmid derived from pACE-1 (33).

### Intracellular MIC assay

Frozen stocks of macrophage THP-1 cells (ATCC TIB-202) were thawed in RPMI-1640 medium (Sigma) supplemented with 10% fetal bovine serum (FBS) (Gibco), 2-mM L-glutamine (Sigma), and 1-mM sodium pyruvate (Sigma). THP-1 cells were passaged only five times and maintained without antibiotics between 2 and 10 × 10^5^ cells/mL at 37°C in a humidified, 5% CO_2_ atmosphere. THP-1 cells (3 × 10^8^) were simultaneously differentiated with phorbol myristate acetate (40 ng/mL, Sigma) and infected for 4 h at a multiplicity of infection of 1:1 with a single cell suspension of H37Rv-Luc. After incubation, infected cells were washed four times to remove extracellular bacilli and resuspended (2 × 10^5^ cells/mL) in RPMI medium supplemented with 10% FBS (Hyclone), 2-mM L-glutamine, and pyruvate and dispensed in white, flat-bottomed 384-well plates (Greiner) in a final volume of 50 µL (max. 0.5% DMSO). Plates were incubated for 5 days under 5% CO_2_ atmosphere at 37°C and 80% relative humidity before growth assessment. The Bright-Glo Luciferase Assay System (Promega, Madison, WI, USA) was used as cell growth indicator for the H37Rv-Luc strain. Luminescence was measured in an Envision Multilabel Plate Reader (PerkinElmer) using the opaque 384-plate Ultra Sensitive luminescence mode, with a measurement time of 50 ms. A 90% reduction in light production was considered growth inhibition, and the IC_90_ value was interpolated from the dose-response curve.

### Extracellular MIC assays

MICs against the H37Rv strain were determined by broth dilution assay in Middlebrook 7H9 medium supplemented with 10% ADC. After incubating at 37°C for 6 days, 25-µL resazurin solution (one tablet in 30-mL sterile PBS) was added to each well. Following incubation at 37°C for two additional days, the lowest concentration of drug that inhibited 90% of resazurin conversion compared to internal DMSO control wells with no drug added was used to define MIC values.

MICs against clinical isolates of *M. tuberculosis* were determined using the mycobacteria growth indicator tubes (MGIT) system. Approximately 1-mg wet weight from a Lowenstein-Jensen slant, with an estimated bacterial load of 10^8^ CFU/mL, was inoculated into McCartney vials containing 1 mL of distilled water and five glass beads. The mixtures were homogenized by vortexing for 1–3 min. The opacity of the suspensions was adjusted by the addition of sterile distilled water to that of a 0.5-McFarland turbidity standard. One hundred microliters was used to inoculate MGIT vials containing serial dilutions of the compounds. MIC values were defined using the BACTEC MGIT 960 System (Becton Dickinson) and following the manufacturer’s instructions.

### HepG2 cytotoxicity assay

HepG2 cells were cultured using Eagle’s minimum essential media (EMEM) supplemented with 10% heat-inactivated FBS, 1% non-essential amino acid (NEAA), and 1% penicillin/streptomycin. Prior to addition of the cell suspension, 250 nL of test compounds per well was pre-dispensed in tissue culture-treated black clear-bottomed 384-well plates (Greiner, cat. no. 781091) with an Echo 555 instrument. After that, 25 µL of HepG2 (cat. no. ATCC HB-8065) cells (∼3,000 cells/well) grown to confluency in EMEM supplemented with 10% heat-inactivated FBS, 1% NEAA, and 1% penicillin/streptomycin were added to each well with the reagent dispenser. Plates were allowed to incubate at 37°C with 20% O_2_ and 5% CO_2_ for 48 hrs. After incubation, the plates were equilibrated to room temperature before ATP levels were measured with the CellTiter Glo kit (Promega) as the cell viability read-out. 25 µL of CellTiter Glo substrate dissolved in the buffer was added to each well. Plates were incubated at room temperature for 10 min for stabilization of luminescence signal and read on a View Lux luminometer with excitation and emission filters of 613 and 655 nm, respectively. The Tox_50_ value corresponds to the concentration of the compound necessary to inhibit 50% of cell growth.

### Cell health assay

This is a three-parameter automated imaging cell-based assay to measure the cytotoxic effect of compounds in human liver-derived HepG2 cells. Using fluorescent staining, we measured the following key parameters in this assay: nuclear size, mitochondrial membrane potential and plasma membrane permeability. HepG2 cells (ATCC HB-8065) were incubated with the test compounds in 384-well plates. After 48 h, the staining cocktail was added. Hoechst 33342 was used to stain nuclei and quantify changes in nuclear morphology. Tetramethylrhodamine, methyl ester is a cationic dye that accumulates in healthy mitochondria that maintain a mitochondrial membrane potential and leaks out of mitochondria when the mitochondrial membrane potential is dissipated. TOTO-3 iodide labels nuclei of permeabilized cells and is used to measure plasma membrane permeability. Following 45 min of incubation with these stains, the plates were sealed using a black seal for reading on an INCell Analyzer 2000 (GE Healthcare). Each parameter measurement produces the percentages of cells which are “live” or “dead.” The IC_50_ is defined as the compound concentration that inhibits 50% of cell growth.

### Ames assay

The Ames assay was carried out as previously described (34) using all strains.

### hERG assay

hERG activity was measured as previously described (35).

### Hepatic microsome stability

Human and animal microsomes and compounds were pre-incubated at 37°C prior to addition of NADPH to final protein concentration of 0.5 mg/mL and a final compound concentration of 0.5 µM. Quantitative analysis was performed using specific liquid chromatography-tandem mass spectrometry (LC-MS/MS) conditions. The half-life, elimination rate constant, and intrinsic clearance (mL/min/g tissue) were determined. The well-stirred model was used to translate to *in vivo* CI values (mL/min/kg).

### *In vivo* pharmacokinetics analysis

Single-dose pharmacokinetics experiments were performed in female C57BL/6 mice, 21–29 g, obtained from Charles River Laboratories (Wilmington, MA, USA) and housed in cages in groups of three animals with water and food *ad libitum*. Animals were maintained for 1 week before the experiment.

The compound was dissolved in 20% Encapsin (Sigma-Aldrich), 5% DMSO (Sigma-Aldrich) in saline solution (Sigma-Aldrich) for intravenous administration, and in 1% methylcellulose (Sigma-Aldrich) in water for oral administration.

For PK analysis, 25 µL of tail blood was collected by microsampling at 0.08, 0.25, 0.5, 1.0, 2.0, 4.0, 6.0, 8.0, and 24.0 h for intravenous pharmacokinetics and 0.25, 0.5, 1.0, 2.0, 4.0, 6.0, 8.0, and 24.0 h for oral pharmacokinetics.

### Assessment of acute efficacy in murine TB models

Specific pathogen-free, 8- to 10-week-old (18–20 g) female C57BL/6 mice were purchased from Harlan Laboratories and were allowed to acclimate for 1 week. The experimental design for the acute assay has been previously described (19). In brief, mice were intratracheally infected with 100,000 CFU/mouse of *M. tuberculosis* H37Rv. Compounds were administered daily for 8 consecutive days starting 24 h after infection. Lungs were harvested on day 9. All lung lobes were aseptically removed, homogenized, and frozen. Homogenates were thawed and plated on 7H11 medium supplemented with 10% OADC plus 0.4% activated charcoal to reduce the effects of compound carryover. CFUs were counted after 18 days of incubation at 37°C. Log_10_ CFU vs dose data were plotted. A sigmoidal dose-response curve was fitted and used to estimate ED_99_ and ED_max_. Data were analyzed using GraphPad software (Prism). The equation is described below. The ED_99_ was defined as the dose in milligram per kilogram that reduced the number of CFUs in the lungs of treated mice by 99% compared to untreated infected mice. The EDmax is the dose in milligram per gram that resulted in 90% of predicted maximal effect.

The *Y* = (log_10_[CFU in the lungs of mice at 24 h after last drug administration]) vs *X*= (the dose level administered per day) are fitted to a four-parameter logistic equation as defined below:


F=99logEC50=logECF−(1/HillSlope)×log(F/(100−F))Y=bottom+(top−bottom)/(1+10((X−logEC50)×HillSlope)).

This equation is a four-parameter logistic equation where “bottom” is the *Y* value at the bottom plateau and “top” is the *Y* value at the top plateau. LogED50 is the *X* value when the response is halfway between bottom and top. “HillSlope” describes the steepness of the curve.

### Modeling and simulations

The calculated exposures at ED_max_ for GSK138 and GSK693 were obtained using the intravenous mouse PK profiles fitting to a bicompartmental model to obtain those parameters to simulate the oral whole blood exposures at ED_max_. Additionally, a monocompartmental model was used to fit the experimental oral pharmacokinetic studies in non-infected mouse together with measured plasma concentrations obtained from the sparse sampling in the experiment 2 in infected mice. Parameters obtained from this fitting were used to simulate the profile after GSK693 administration at 100, 200, and 300 mg/kg in the combination study and to calculate the associated exposures (see Table 2).

### Blood and plasma pharmacokinetic sampling and analysis

#### Blood sample collection from non-infected mice (PK studies)

Blood samples (25 µL) were taken from the lateral tail vein using a micropipette and were mixed, vortexed with 25 µL of saponin 0.1%, and frozen at −80°C until analysis.

#### Plasma sample collection from infected mice

Tail vein blood was collected at the indicated time points. Briefly, an incision was made in the lateral tail vein. Blood (20–50 µL) was collected in BD Microtainer PST lithium heparin tubes from each mouse. The tubes were kept on ice before being centrifuged at 8,000 rpm for 5 min. The supernatant plasma (15–30 µL) was transferred to labeled microcentrifuge tubes, frozen, and stored at −80°C and then shipped on dry ice to GSK for further analysis.

#### Sample pre-treatment and LC-MS/MS analysis

Ten microliters of plasma or blood samples thawed at ambient temperature was mixed with 200 µL of acetonitrile (ACN):MeOH (80:20). After this protein precipitation step, samples were filtered using a 0.45-µm filter plate [Multiscreen Solvinert 0.45-μm polytetrafluoroethylene (PTFE), Millipore] and then filtered using a 0.2-µm filter [AcroPrep Advance 96 Filter Plate 350 µL, 0.2-µm polytetrafluoroethylene (PTFE)] to ensure sterilization prior to LC-MS analysis.

An Acquity ultra-performance liquid chromatography (UPLC) system (Waters Corp., Milford, MA, USA) coupled to a triple quadrupole mass spectrometer (API 4000; AB Sciex, Foster City, CA, USA) was used for the analysis.

The chromatographic separation was conducted at 0.4 mL/min in an Acquity UPLC BEH C18 column (50 × 2.1 mm i.d., 1.7 mm; Waters Corp.) at 40°C with ACN (Sigma-Aldrich) and 0.1% formic acid as eluents.

Sciex Analyst software was used for the data analysis. The non-compartmental data analysis was performed with Phoenix WinNonlin software in order to determine pharmacokinetic parameters and exposure.

### High-dose aerosol mouse infection model

Female specific pathogen-free BALB/c mice, aged 5–6 weeks, were purchased from Charles River. Mice were infected by aerosol using the Inhalation Exposure System (Glas-col, Terre Haute, IN, USA) using a log phase culture of *M. tuberculosis* H37Rv with an OD_600_ of 0.8–1.0 to implant approximately 3.5–4.0 log_10_ CFU in the lungs. Treatment started 2 weeks later (D0). Mice were sacrificed for lung CFU counts the day after infection and on D0 to determine the number of CFU implanted and the number present at the start of treatment, respectively.

### Antibiotic treatment

Mice were treated with the drugs and drug combinations indicated in Fig. 2 through 6 at the following doses (in mg/kg body weight): bedaquiline (25), pretomanid (50 or 100), moxifloxacin (100), linezolid (100), isoniazid (10), rifampicin (10), sutezolid (50), GSK138 (50, 100, or 200), GSK693 (100, 200, or 300), GSK656 (10), GSK286 (50), NITD-113 (150), and pyrazinamide (150). GSK693, GSK138, and GSK286 were formulated in 1% methylcellulose solution. GSK656 was formulated in distilled water. Other drugs were formulated as previously described (36–38). Bedaquiline and pretomanid were administered in back-to-back gavages and separated from companion drugs by at least 3 h. Rifampicin was administered alone at least 1 h before any companion drug.

### Evaluation of drug efficacy

Efficacy determinations were based on lung CFU counts after 4 or 8 weeks of treatment and, in one experiment, cohorts of mice were also kept for 12 weeks after completing 8 or 12 weeks of treatment to assess for relapse-free cure. At each time point, lungs were removed aseptically and homogenized in 2.5 mL of PBS. Serial 10-fold dilutions of lung homogenate were plated on selective 7H11 agar plates. To assess for relapse-free cure, the entire lung homogenate was plated. In experiments with bedaquiline, lung homogenates were plated on 7H11 agar supplemented with 0.4% activated charcoal to reduce drug carryover, and the concentrations of selective antibiotics in the media were doubled to mitigate binding to charcoal.

### Statistical analysis

Group means were compared by one-way analysis of variance with Dunnett’s correction for multiple comparisons or by Student’s *t*-test, as appropriate, using GraphPad Prism version 8.
